# Clinical Implications of Ki‐67 Index, Grade and Hormonal Changes in Pancreatic Neuroendocrine Tumors: Insights Into Tumor Heterogeneity Based on Primary and Secondary Lesions

**DOI:** 10.1002/jhbp.70028

**Published:** 2025-11-16

**Authors:** Masatoshi Murakami, Nao Fujimori, Kazuhide Matsumoto, Keijiro Ueda, Kohei Nakata, Masafumi Nakamura, Takeo Yamamoto, Yoshinao Oda, Tetsuhide Ito, Yoshihiro Ogawa

**Affiliations:** ^1^ Department of Medicine and Bioregulatory Science, Graduate School of Medical Sciences Kyushu University Fukuoka Japan; ^2^ Department of Surgery and Oncology, Graduate School of Medical Sciences Kyushu University Fukuoka Japan; ^3^ Department of Anatomic Pathology, Pathological Sciences, Graduate School of Medical Sciences Kyushu University Fukuoka Japan; ^4^ Neuroendocrine Tumor Centre Fukuoka Sanno Hospital Fukuoka Japan; ^5^ Department of Gastroenterology, Graduate School of Medical Sciences International University of Health and Welfare Fukuoka Japan

**Keywords:** grade change, hormonal phenotype, metastasis and recurrence, pancreatic neuroendocrine tumors, tumor heterogeneity

## Abstract

**Background:**

Pancreatic neuroendocrine tumors (PanNETs) show heterogeneity, including temporal shifts in proliferation and hormone production; however, their clinical implications remain uncertain.

**Methods:**

This retrospective study included 114 patients with metastatic or recurrent PanNETs at Kyushu University Hospital. Paired specimens from 46 patients (27 synchronous metastases and 19 recurrences) were evaluated for Ki‐67 index and tumor grade. Proliferation change was defined as grade progression or a ≥ 10% absolute Ki‐67 increase. Hormonal phenotype changes were assessed in all patients.

**Results:**

In metastases, mean Ki‐67 increased from 12.3% to 16.4% (*p* = 0.0043); 22.2% showed a ≥ 10% increase, and 33.3% progressed in grade. In recurrences, Ki‐67 increased from 8.8% to 9.3% (*p* = 0.8256); 15.8% showed a ≥ 10% increase, and 21.1% progressed in grade. Median progression‐free survival was 7.8 months in metastases and 17.1 months in recurrences. Median overall survival was significantly longer in the recurrence group (124.8 vs. 32.5 months, *p* = 0.003). Hormonal transformation occurred in six patients (5.3%), mostly during progressive hepatic disease.

**Conclusion:**

A subset of PanNETs exhibited increased proliferation of metastases or recurrence without detrimental survival effects, possibly because of timely treatment adjustments. Rebiopsy may be useful for detecting proliferative changes, whereas hormonal shifts highlight tumor heterogeneity and warrant continued clinical and biochemical monitoring.

## Graphical abstract

In this retrospective study of metastatic and recurrent pancreatic neuroendocrine tumors, Murakami and colleagues demonstrated frequent discordance in Ki‐67 index and World Health Organization grade between primary and secondary lesions. Increased proliferation did not compromise survival, likely reflecting treatment adaptation. Rare hormonal phenotype shifts further underscore the importance of biochemical monitoring.

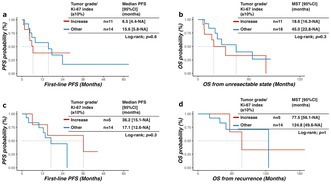

## 1 Introduction

Pancreatic neuroendocrine tumors (PanNETs) are heterogeneous neoplasms in which the Ki‐67 index is a key determinant of histological grade and treatment selection [1, 2]. Tumor grade at diagnosis is one of the most powerful predictors of outcome, with prior studies showing 5‐year overall survival (OS) rates of approximately 89% for G1, 48% for G2, and nearly 0% for the overall G3 cohort that included both well‐differentiated NET G3 and poorly differentiated neuroendocrine carcinomas (NEC) [3]. Among these, well‐differentiated NET G3 demonstrates intermediate outcomes—worse than NET G1/G2 but better than NEC—with median overall survival of approximately 22 months in unresectable or metastatic cases [4] and 54.0 months in resected cases [5]. Consequently, the Ki‐67 index is routinely used in pathology reporting and clinical decision‐making to guide therapy selection [2, 5]. However, substantial intratumoral and intertumoral heterogeneity can manifest as either spatial (differences between primary and metastatic sites) or temporal (evolution over time) changes in the Ki‐67 index or World Health Organization (WHO) grade [6, 7, 8, 9], potentially altering the prognosis and therapeutic strategy. Whether to perform a rebiopsy in the setting of disease progression remains an important clinical question. However, the prognostic and therapeutic implications of such changes are not fully understood.

Another under‐recognized aspect of PanNET behavior is the potential for hormonal phenotypic changes over the course of the disease. PanNETs can be classified as functional (hormone‐secreting, causing clinical syndromes) or non‐functional. Few case reports have documented non‐functional NETs that later began secreting hormones, or functional tumors that switched or added new hormonal secretions as they advanced [10, 11, 12]. Although this phenomenon is occasionally encountered in clinical practice, its frequency, patterns, and impact remain poorly understood.

This study sought to evaluate intrapatient biological heterogeneity in PanNETs by assessing two distinct but potentially interrelated features: proliferation changes, defined by either WHO grade progression or an absolute Ki‐67 increase of ≥ 10%, and hormonal phenotype transformation. The ≥ 10% threshold was chosen because PanNETs with higher Ki‐67 within the same WHO grade—particularly within the G2 range—are increasingly recognized as having more aggressive clinical behavior, suggesting that reliance on grade categories alone may underestimate clinically relevant changes [13, 14]. By analyzing these parameters in paired primary and secondary lesions, this study aimed to clarify their frequency, clinical significance, and implications for patient management.

## 2 Methods

### 2.1 Patients and Data Collection

**FIGURE 1:**
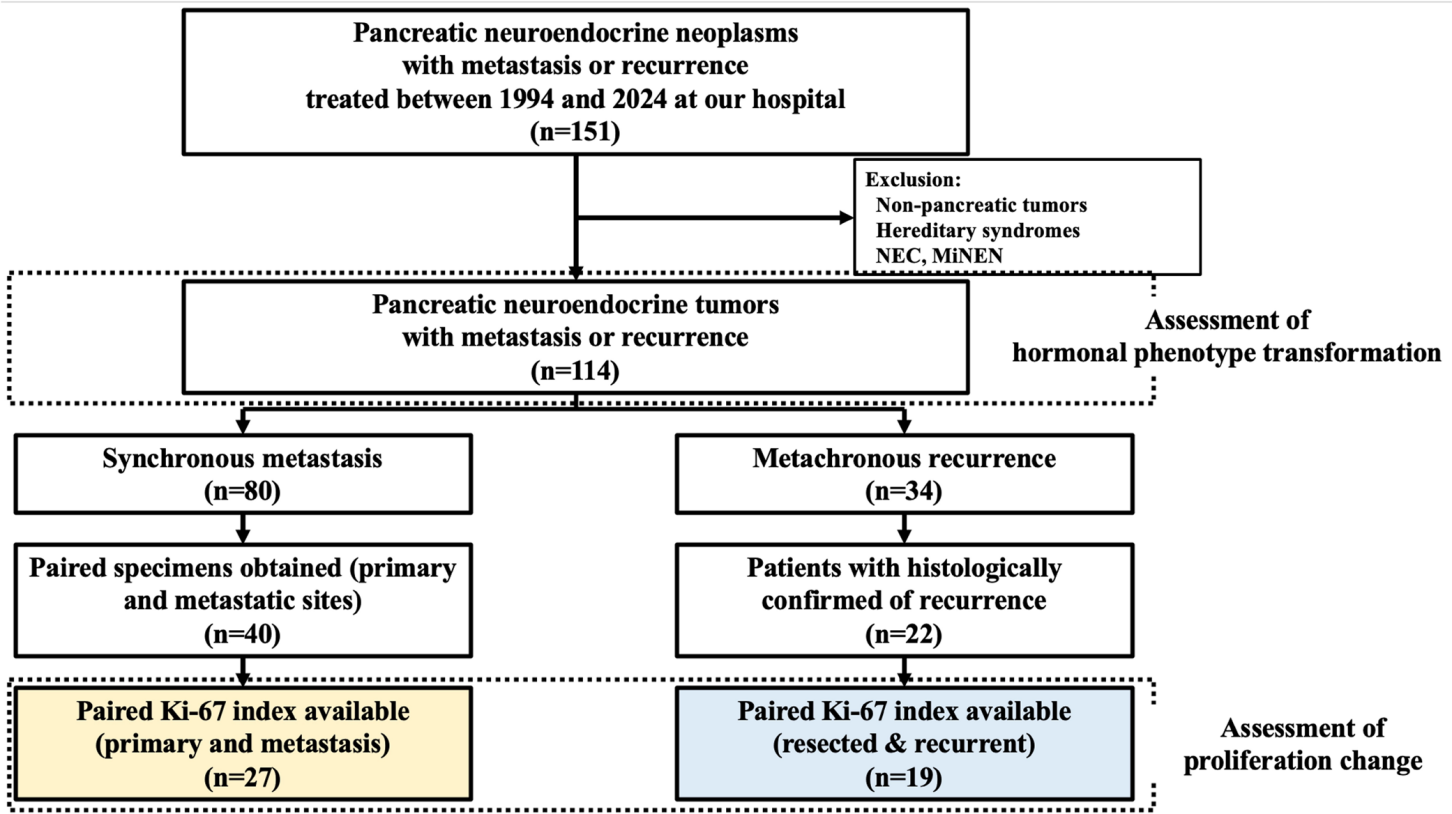
Patient selection and subgroup classification flowchart.

The retrospective study analyzed patients with PanNETs who were treated at Kyushu University Hospital between January 1994 and December 2024. Among the 151 patients with metastatic or recurrent pancreatic neuroendocrine neoplasms, those with non‐pancreatic primary sites, genetic syndromes, poorly differentiated neuroendocrine carcinomas, or mixed neuroendocrine‐non‐neuroendocrine neoplasms were excluded, leaving 114 eligible patients. Hormonal phenotypic shifts were evaluated in all 114 patients. Within this group, 62 patients with histologically confirmed metastases or recurrence were identified, and among them, 46 patients with paired primary and secondary tumor specimens available for Ki‐67 assessment (27 synchronous metastases and 19 metachronous recurrences) were analyzed (Figure 1).

This study was approved by the Institutional Review Board of Kyushu University Hospital (#23023‐00), and the requirement for informed consent was waived because of retrospective data collection. Previously published case reports of VIPoma [15] and ACTHoma [16] were incorporated into this broader analysis of hormonal phenotypic changes.

### 2.2 Definitions

All tumor samples were reviewed by experienced pathologists. The Ki‐67 index was assessed by manually counting as many cells as feasible (typically 500–2000 cells) in areas with the highest labeling [6]. The tumors were graded according to the 2019 WHO classification [1].

Change in proliferation was defined as either WHO grade progression or an absolute Ki‐67 index increase of ≥ 10% between the primary and secondary lesions. The hormonal phenotype was classified as functional or non‐functional based on the presence of characteristic hormonal symptoms with elevated serum hormone levels (above the upper normal limit) and/or positive immunohistochemistry for the corresponding hormone. Hormonal phenotype transformation was defined as a change in the functional status or acquisition of new hormones during the disease course.

OS was defined as the primary endpoint, representing the interval from diagnosis (metastatic group) or recurrence (recurrent group) to death or the last follow‐up. Progression‐free survival (PFS) was defined as a supportive endpoint, defining the interval from initiation of first‐line (metastatic) or first post‐recurrence (recurrent) systemic therapy to disease progression or death.

### 2.3 Statistical Analysis

Continuous variables are summarized as medians with interquartile ranges, and categorical variables as counts and percentages. Wilcoxon rank‐sum test and Fisher's exact test were used for comparison. Paired comparisons of Ki‐67 indices were performed using paired *t*‐tests or Wilcoxon signed‐rank tests, and WHO grade shifts were analyzed using McNemar's test. Survival analyses were conducted using the Kaplan–Meier method and compared using the log‐rank test. Statistical significance was defined as a *p*‐value < 0.05. All analyses were performed using R version 4.5.0.

## 3 Results

### 3.1 Patient Characteristics

Forty‐six patients with PanNETs and paired primary‐secondary tumor specimens were included (Figure 1): 27 with synchronous metastases (metastatic group) and 19 with recurrence after curative resection (recurrent group). The clinical characteristics are summarized in Table 1.

**TABLE 1:** Clinical and pathological characteristics of included patients.

Characteristics	Metastatic group (*n* = 27)	Recurrent group (*n* = 19)
Age at diagnosis, median (range)	58.5 (21–86)	55.8 (38–82)
Sex		
Man	15 (55.6)	7 (36.8)
Women	12 (44.4)	12 (63.2)
Functionality		
Functioning	5 (18.5)	3 (15.8)
Non‐functioning	22 (81.5)	16 (84.2)
Primary tumor location		
Pancreatic head	10 (37.0)	10 (52.6)
Pancreatic body	2 (7.4)	2 (10.5)
Pancreatic tail	14 (51.9)	7 (36.8)
Diffuse	1 (3.7)	0 (0)
Primary tumor size, median (range) (mm)	36.0 (10.0–130.0)	34.0 (12.0–56.0)
WHO Grade (at diagnosis)^a^		
NET G1	2 (7.4)	6 (31.6)
NET G2	16 (59.3)	12 (63.2)
NET G3	9 (33.3)	1 (5.3)
Stage^b^ (at diagnosis)		
Stage I	0 (0)	2 (10.5)
Stage II	0 (0)	6 (31.6)
Stage III	0 (0)	7 (36.8)
Stage IV	27 (100)	3 (15.8)
Not available	0 (0)	1 (5.3)
Metastatic/recurrent site^c^		
Liver	27	16
Lymph node	6	5
Peritoneum	2	0
Bone	2	0
Other	2	0
Pancreas	0	2
Specimen acquisition method (primary)		
EUS‐TA	21 (80.8)	0 (0)
Resection	4 (14.8)	19 (100)
Percutaneous biopsy	1 (3.7)	0 (0)
Endoscopic biopsy (gastric invasion)	1 (3.7)	0 (0)
Specimen site (metastasis/recurrence)^b^		
Liver	27 (100)	17 (89.5)
Lymph node	0 (0)	2 (10.5)
Pancreas	0 (0)	2 (10.5)
Stomach	0 (0)	1 (5.3)
Specimen acquisition method (metastasis/recurrence)^b^		
Percutaneous biopsy (liver metastasis)	22 (81.5)	12 (63.2)
Hepatic metastasectomy	5 (18.5)	3 (15.8)
Lymph node metastasectomy	0 (0)	2 (10.5)
EUS‐TA (liver)	0 (0)	1 (5.3)
EUS‐TA (pancreas)	0 (0)	1 (5.3)
EUS‐TA (lymph node)	0 (0)	1 (5.3)
Pancreatic metastasectomy	0 (0)	1 (5.3)
Gastric metastasectomy	0 (0)	1 (5.3)
1st line treatment		
SSA	4 (14.8)	9 (47.4)
Target therapy ± SSA	12 (44.4)	8 (42.1)
Chemotherapy ± SSA	10 (37.0)	2 (10.5)
Surgery	1 (3.7)	0 (0)
Recurrence free survival, months (median [95% CI])	—	36.6 [28.3–66.8]
Median follow‐up time, months (range)	18.6 (5.3–85.5)	49.6 (5.7–209.2)

Abbreviations: CI, confidence interval; EUS‐TA, endoscopic ultrasound‐guided tissue acquisition; NET, neuroendocrine tumor; SSA, somatostatin analogs.

^a^ For the metastatic group, tumor grade was based on findings at diagnosis, while for the recurrent group, tumor grade was determined from the resected specimen.

^b^ The 8th edition of the Union for International Cancer Control tumor‐node‐metastasis classification.

^c^ Overlapping.

The metastatic group more frequently had pancreatic tail primary tumors and a higher baseline WHO grade. The proportion of NET G3 was 33.3% and 5.3% in the metastatic and recurrent groups, respectively. Liver involvement was universal in the metastatic group, whereas 84.2% of the patients with recurrence had liver involvement. Curative resection was performed in all patients with recurrence, but in only four (14.8%) of the patients with metastases. The median follow‐up duration was longer in the recurrent group (49.6 months) than in the metastatic group (18.6 months).

**FIGURE 2:**
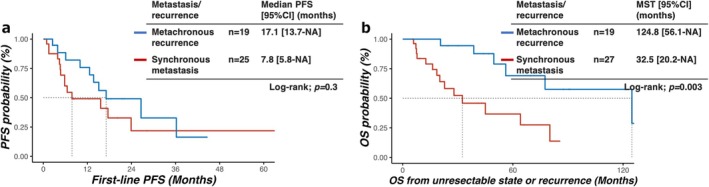
Survival outcomes in the metastatic group vs. the recurrent group. (a) Progression‐free survival and (b) overall survival stratified by disease presentation (metastatic vs. recurrent). In the metastatic cohort, two patients underwent curative resection and did not receive systemic therapy; these patients were excluded from the PFS analysis (*n* = 25) but included in the OS analysis (*n* = 27).

When comparing overall outcomes between groups, the recurrent group had significantly longer OS (median OS 124.8 months [56.1–NA]) than the metastatic group (32.5 months [20.2–NA], *p* = 0.003), although median PFS was not statistically different (17.1 months [13.7–NA] vs. 7.8 months [5.8–NA], *p* = 0.2) (Figure 2). In the metastatic cohort, two patients underwent curative resection and remained disease‐free without receiving systemic therapy; because PFS was defined from initiation of systemic therapy, these patients were excluded from the PFS analysis (*n* = 25 for PFS curves), whereas all 27 metastatic patients were included in the OS analysis.

### 3.2 Changes in the Ki‐67 Index and WHO Grade Between Primary and Secondary Lesions

In the metastatic group, the mean Ki‐67 index increased from 12.3% in primaries to 16.4% in metastases (*p* = 0.0043) (Figure S1a and Table 2), with a ≥ 10% absolute increase in Ki‐67 index in six of 27 patients (22.2%). Overall, 59.3% showed an increase, whereas five patients (18.5%) showed a decrease.

**TABLE 2:** Comparison of the Ki‐67 index between primary and metastatic/recurrent lesions.

Parameter	Primary lesion	Metastatic lesion	*p*
Ki‐67 index, mean ± SD (%)	12.3 ± 13.4	16.4 ± 15.5	0.0043
Ki‐67 index, median (IQR) (%)	5.0 (3.5, 16.5)	10.0 (5.0, 26.4)	
Comparison of Ki‐67 Index, *n* (%)			
Increased in metastasis	—	16 (59.3)	
Unchanged	—	6 (22.2)	
Decreased in metastasis	—	5 (18.5)	
Grade increase, *n* (%)			
Upgrade in metastasis	—	9 (33.3)	
Unchanged	—	17 (63.0)	
Downgrade in metastasis	—	1 (3.7)	
Ki‐67 index, mean ± SD (%)	8.8 ± 8.7	9.3 ± 6.7	0.8256
Ki‐67 index, median (IQR) (%)	6.0 (1.0, 15.0)	8.0 (3.0, 15.0)	
Comparison of Ki‐67 Index, *n* (%)			
Increased in recurrence	—	7 (36.8)	
Unchanged	—	5 (26.3)	
Decreased in recurrence	—	7 (36.8)	
Grade increase, *n* (%)			
Upgrade in recurrence	—	4 (21.1)	
Unchanged	—	12 (63.2)	
Downgrade in recurrence	—	3 (15.8)	

Abbreviations: IQR, interquartile range; SD, standard deviation.

In the recurrent group, the Ki‐67 indices remained stable on average (8.8% vs. 9.3%, *p* = 0.8256) (Figure S1b and Table 2). A ≥ 10% increase in Ki‐67 index was observed in three of 19 patients (15.8%). The overall trends were more variable: 36.8% of patients showed an increase, 26.3% showed a decrease, and 36.8% remained stable.

WHO grade shifts (Figure S1c,d; Table 2; Table S1) occurred in 33.3% of the patients with metastases (most often G1 → G2 or G2 → G3) versus 21.1% of the patients with recurrence (all G1 → G2, no G2 → G3). In the metastatic group, none of the tested variables significantly predicted an increase in cell proliferation (Table S2). In the recurrent group, G2 or G3 primary tumors were significantly associated with a lower risk of increased proliferation (OR 0.04, *p* = 0.019), most likely because higher‐grade tumors have limited potential for additional upward grade shifts (Table S3).

Because most Ki‐67 assessments in the metastatic group were derived from biopsy specimens, whereas resected samples were used in the recurrent group, these methodological differences should be considered when interpreting the comparison of Ki‐67 indices between groups.

### 3.3 Survival Outcomes by Proliferation Change

**FIGURE 3:**
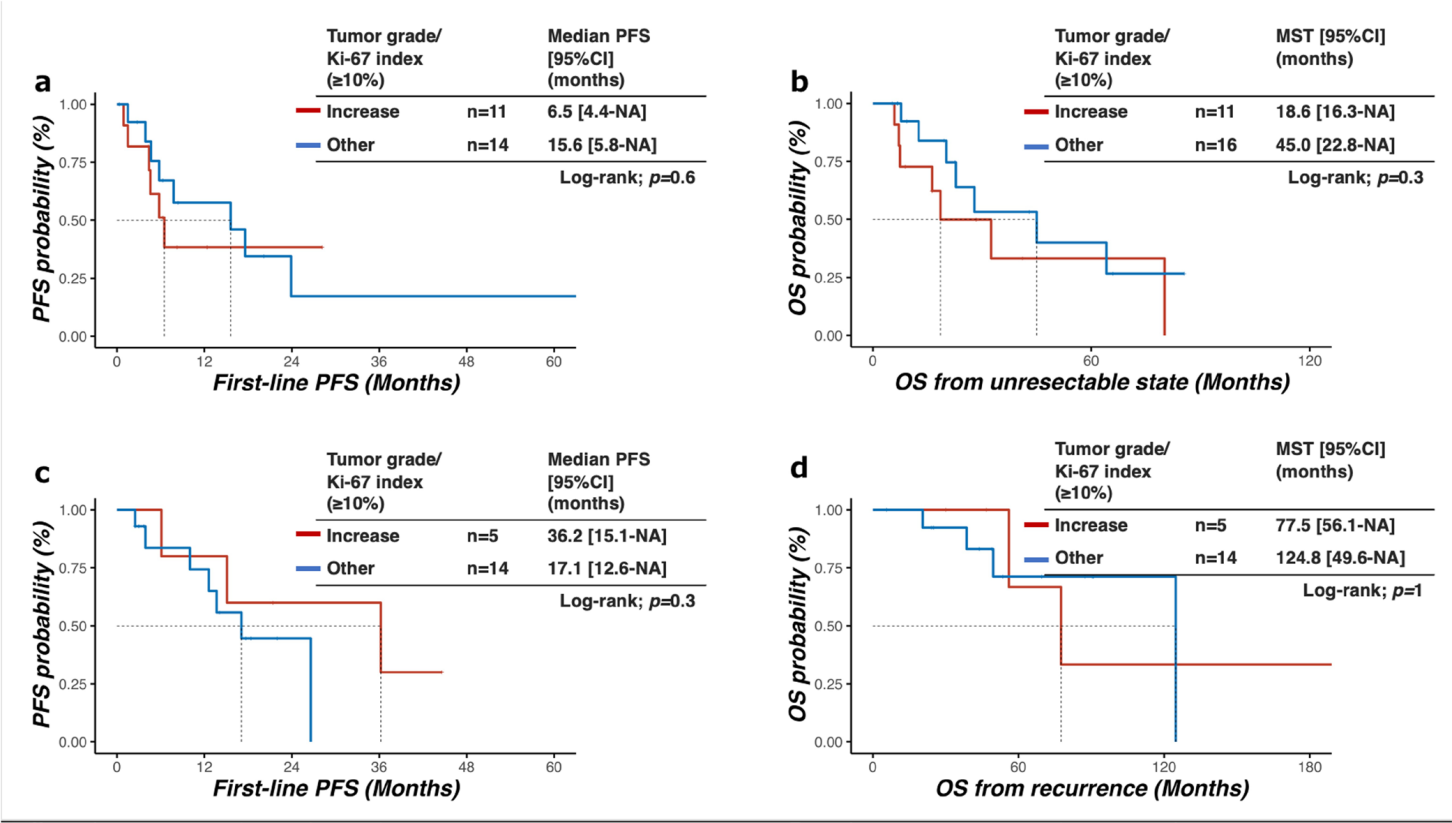
Survival outcomes stratified by Ki‐67 index or WHO grade increase. (a) Progression‐free survival and (b) overall survival in the metastatic group stratified by presence vs. absence of Ki‐67 or grade increase. (c) Progression‐free survival and (d) post‐recurrence survival in the recurrent group stratified by presence vs. absence of Ki‐67 or grade increase. CI, confidence interval; MST, median survival time; NA, not available; OS, overall survival; PFS, progression‐free survival. In the metastatic cohort, two patients underwent curative resection and did not receive systemic therapy; these patients were excluded from the PFS analysis (*n* = 25) but included in the OS analysis (*n* = 27).

Survival analyses stratified by increased proliferation are shown in Figure 3. In the metastatic group, Ki‐67 expression and grade increase were not significantly associated with poor OS (*p* = 0.3) or PFS (*p* = 0.6). Similarly, in the recurrent group, post‐recurrence OS and PFS did not differ significantly according to the proliferation status (*p* = 1 and 0.3, respectively).

The OS advantage of the recurrent group compared with the metastatic group (Figure 2) persisted regardless of the proliferation change status, suggesting that the baseline disease extent exerts a stronger influence on long‐term outcomes than changes in Ki‐67 or grade alone.

### 3.4 Hormonal Phenotype Transformation

**FIGURE 4:**
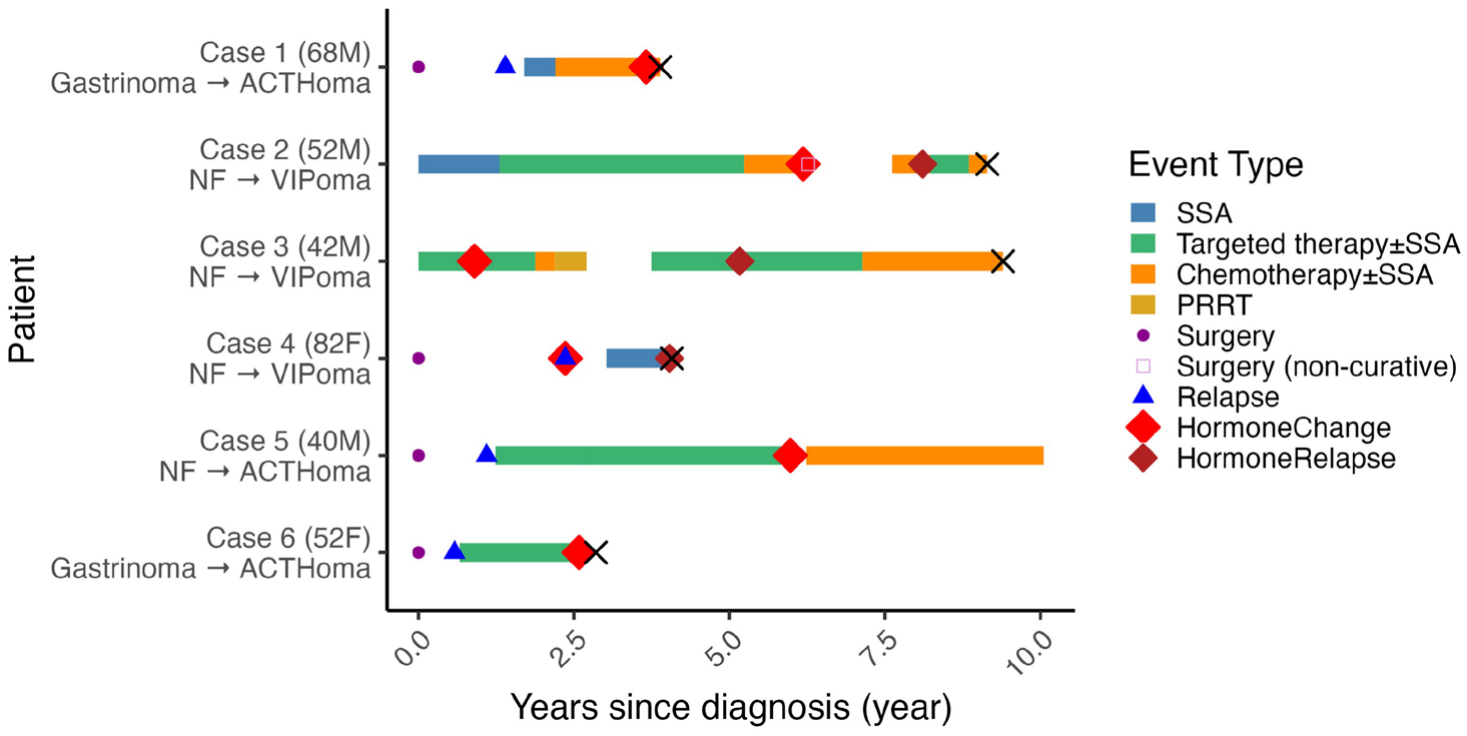
Timeline of hormonal phenotype transformation in six PanNET patients. Each row represents an individual patient with functional transformation during the clinical course. The colored bars indicate the duration and type of systemic therapy used. Diamonds indicate the emergence of new hormonal syndromes (VIPoma and ACTHoma). The cross‐symbols indicate death. This figure illustrates the delayed onset of hormonal shifts frequently observed in advanced disease with liver‐dominant progression. ACTHoma, adrenocorticotropic hormone‐producing tumor; NF, non‐functioning tumor; PanNET, pancreatic neuroendocrine tumor; PRRT, peptide receptor radionuclide therapy; SSA, somatostatin analog; VIPoma, vasoactive intestinal peptide‐producing tumor.

Across the entire cohort (*n* = 114), six patients (5.3%) exhibited hormonal phenotype changes during the disease course (Table 3 and Figure 4). Four patients with initially non‐functional tumors became functional (three VIPomas, one ACTHoma), and two gastrinomas acquired ACTH production. These changes occurred years after diagnosis, often during progressive hepatic disease and after multiple systemic therapies. These shifts highlight the dynamic clinical behavior of PanNETs and the importance of ongoing biochemical and clinical surveillance.

**TABLE 3:** Clinical characteristics of PanNET patients exhibiting hormonal phenotype switching.

Case	Age	Sex	Tumor location	Primary tumor functionality	Hormonal phenotype change	Time to hormonal change (months)	Disease stage at initial diagnosis	Disease status at time of hormonal change	Ki‐67 index at primary lesion (%)	Ki‐67 index at metastatic lesion (%)	Ki‐67 index at recurrent lesion (%)
1	68	M	Pancreatic head	Gastrinoma	ACTHoma	43.8	Local disease	Liver recurrence	12.8	—	Not‐evaluated
2	52	M	Pancreatic tail	Non‐functioning	VIPoma	81.5	Liver metastasis	Liver metastasis	8.0	Not‐evaluated	—
3	42	M	Pancreatic tail	Non‐functioning	VIPoma	11.0	Liver metastasis	Liver metastasis	1.0	Not‐evaluated	—
4	82	F	Pancreatic tail	Non‐functioning	VIPoma	28.3	Local disease	Local recurrence	17.0	—	1.0
5	40	M	Pancreatic tail	Non‐functioning	ACTHoma	71.6	Local disease	Liver recurrence	3.0	—	Not‐evaluated
6	52	F	Pancreatic tail	Gastrinoma	ACTHoma	30.3	Liver metastasis	Liver recurrence	6.5	7.7	10.0

Case	Symptoms at time of hormonal change	Treatment ongoing at time of hormonal change	Treatment modification after hormonal change	Outcome	OS from initial diagnosis (months)	OS from hormonal change (months)
1	moon face appearance, truncal obesity, severe pitting edema, increased neutrophilia, hypokalemia, worsened glucose tolerance	SSA + Everolimus	Corticosteroid synthesis inhibitors	Dead of hormone‐related complications	46.6	2.7
2	severe watery diarrhea, dehydration, hypokalemia, muscle weakness, hypotension	SSA + TS1	Continuous intravenous infusion of SSA Bariatric surgery	Dead of hormone‐related complications	94.0	12.5
3	severe watery diarrhea, dehydration, hypokalemia	Everolimus	SSA PRRT	Dead of cancer	112.7	101.8
4	severe watery diarrhea, dehydration, hypokalemia, muscle weakness	Post‐curative resection surveillance	SSA	Dead of hormone‐related complications	48.8	20.5
5	moon face appearance, truncal obesity, severe pitting edema, petechial bleeding, increased neutrophilia, hypokalemia, hypercalcemia, worsened glucose tolerance	SSA + Everolimus	Corticosteroid synthesis inhibitors SSA + STZ/5‐FU	Alive with disease	95.0	23.4
6	moon face appearance, truncal obesity, pitting edema, increased neutrophilia, hypokalemia, hypercalcemia, worsening glucose tolerance	SSA + Sunitinib	Corticosteroid synthesis inhibitors	Dead of cancer	33.5	3.2

Abbreviations: ACTHoma, adrenocorticotropic hormone‐producing tumor; PanNET, pancreatic neuroendocrine tumor; PRRT, peptide receptor radionuclide therapy; SSA, somatostatin analogs; STZ/5‐FU, streptozotocin/5‐fluorouracil; TS‐1, tegafur–gimeracil–oteracil potassium; VIPoma, vasoactive intestinal peptide‐producing tumor.

## 4 Discussion

This study retrospectively analyzed 46 patients with PanNETs who had both primary and secondary lesions pathologically evaluated to elucidate the clinical significance of intrapatient changes in tumor proliferation. Furthermore, hormonal phenotypic shifts in a broader cohort of 114 patients with metastatic or recurrent disease were assessed. Taken together, our study provides real‐world insights into the biological evolution of PanNETs over the course of the disease and its clinical implications.

Among the 46 patients with paired specimens, approximately one‐third of those with synchronous metastases demonstrated WHO grade progression in the metastatic lesion, and over half showed an increase in Ki‐67 index, with 22.2% showing a ≥ 10% absolute rise. These differences primarily represent spatial heterogeneity, reflecting variations in proliferative activity between distinct tumor sites at a single time point, rather than true temporal evolution. In contrast, changes observed in the recurrent group more accurately reflect temporal heterogeneity, indicating biological evolution over time. This finding supports prior reports that grade discordance between primary and metastatic PanNETs occurs in approximately 20%–30% of patients with PanNETs [17, 18], underscoring the biological divergence that may arise during disease spread. These data highlight that metastatic lesions cannot be assumed to retain the same grade or proliferative activity as the primary tumor and reinforce the clinical value of reassessing tumor biology at the metastatic site. However, because most Ki‐67 evaluations in the metastatic group were based on biopsy samples, intratumoral heterogeneity may not have been fully captured, potentially leading to underestimation of proliferative activity in some cases [6, 8]. In contrast, patients with metachronous recurrence following surgical resection exhibited more heterogeneous patterns, with fewer patients (21.1%) showing grade progression (from G1 to G2). This is somewhat lower than the 34% rate of grade increase reported by Merola et al. in a large multicenter cohort of recurrent GEP‐NETs [19], although their population had a higher proportion of G1 tumors (median Ki‐67, 3%) than ours (median, 6.0%), which may account for this difference.

Importantly, patients with metachronous recurrence were found to have a markedly longer OS than those presenting with synchronous metastases (median 124.8 vs. 32.5 months). To our knowledge, no previous study has directly compared survival between these two clinical scenarios in PanNETs. Worse prognosis in the metastatic group may reflect both a higher burden of disease and a greater prevalence of G3 tumors at presentation. However, whether this distribution truly represents the inherent biological aggressiveness of synchronous metastatic PanNETs or is merely a result of selection bias and the limited sample size of our cohort remains uncertain. This observation underscores that metastatic and recurrent cohorts should not be lumped together for prognostic analyses and suggests that thorough sampling and grading of metastatic lesions are essential for optimal management.

Despite frequent increases in proliferation, no significant differences in OS or PFS were observed between patients with and without an increase in Ki‐67 or grade. This contrasts with prior studies, such as that by Keck et al. which showed worse survival in patients with grade discordance between primary and metastasis [17, 20]. Several factors could explain this discrepancy. First, the sample size was limited; thus, it was underpowered to detect modest differences in survival. Second, therapeutic intensification in response to biopsy‐proven progression likely mitigates any prognostic disadvantages. In our practice, the identification of a higher Ki‐67 index or grade typically triggers early treatment escalation, including the addition of chemotherapy or targeted agents, such as everolimus. All recurrent patients with an increase in Ki‐67 or grade in our cohort received intensified therapy, whereas many patients without such changes remained on somatostatin analogs alone. This adaptive management likely narrowed the survival gap between biologically distinct groups, exemplifying the well‐described “treatment paradox,” a phenomenon in which the identification of a high‐risk feature prompts interventions that reduce its impact on outcomes.

In the overall cohort of 114 patients with metastases or recurrence, our observation of hormonal phenotype transformation in six patients (5.3%) highlights the functional plasticity of PanNETs. These findings warrant further discussion given their implications for disease monitoring and treatment strategies. Phenotypic changes, including the acquisition of ectopic ACTH or VIP secretion, occur late in the disease course, often accompanied by progressive liver metastases and treatment resistance. Previous reports have described similar transitions in advanced NETs, suggesting that subclonal evolution under therapeutic pressure may drive novel hormone production [10, 21]. Importantly, such transformations frequently present as emergent clinical syndromes such as Cushing's syndrome or secretory diarrhea, which require immediate multidisciplinary intervention. This emphasizes the need for continuous monitoring of new hormonal symptoms in patients with previously non‐functional or gastrin‐producing tumors. Moreover, these observations illustrate the profound dimension of tumor heterogeneity, not only in proliferative indices but also in endocrine function, underscoring the complex biology of PanNETs. Further studies are warranted to elucidate the molecular drivers of hormonal switching, which may serve as prognostic markers and potential therapeutic targets. These findings reinforce the importance of longitudinal clinical and pathological assessments, including rebiopsy, in patients with clinical deterioration [21].

Our results emphasize the potential clinical value of rebiopsy for the progression of PanNETs. Updated histopathological assessment may assist in selecting the most appropriate treatment strategy and in optimizing therapeutic decision‐making, even in the absence of radiological progression. Furthermore, vigilance for new hormonal symptoms is warranted throughout follow‐up, as functional changes can arise independently of proliferative changes. Multidisciplinary care involving endocrinologists and oncologists is essential to manage such patients.

The strengths of this study include the availability of paired pathological samples, long‐term follow‐up, and the inclusion of hormonal phenotype data from the entire metastatic/recurrent cohort. However, several limitations should be acknowledged, including the relatively small sample size and potential selection bias (only patients undergoing rebiopsy were included). Systemic therapies for PanNETs evolved substantially over the three decades covered by this study; this treatment‐era heterogeneity may limit the interpretability of PFS over time. While multiple era cut‐offs could be justified, the case distribution across eras was imbalanced, precluding a statistically meaningful stratified analysis. We therefore emphasized OS as a more robust indicator of long‐term outcomes. Larger prospective studies are warranted to validate whether quantitative Ki‐67 changes or hormonal phenotypic shifts can serve as reliable prognostic and treatment markers.

In conclusion, intrapatient increases in Ki‐67 expression and WHO grades occur in a substantial proportion of PanNETs and may reflect the evolution of tumor biology. Moreover, a marked difference was observed in OS between patients with synchronous metastasis and metachronous recurrence, with significantly worse metastatic presentations despite similar treatment modalities. In a broader cohort, hormonal phenotype shifts, although infrequent, further highlight the functional heterogeneity of these tumors. The absence of a survival impact in our study likely reflects timely treatment adaptations based on the updated pathological findings. These results suggest that rebiopsy of progressive lesions may aid in optimizing treatment strategies and underscore the importance of continued clinical vigilance for new hormonal symptoms. Larger prospective studies are warranted to validate the prognostic and therapeutic implications of quantitative changes in Ki‐67 expression and functional transformations.

## Ethics Statement

Approval of the research protocol by an Institutional Review Board. This study was approved by the Institutional Review Board of Kyushu University Hospital (#23023‐00).

## Consent

The review board waived the requirement for informed consent because of retrospective data collection.

## Conflicts of Interest

The authors declare no conflicts of interest.

## Supporting information


**Figure S1:** Paired changes in Ki‐67 index and WHO grade between primary and secondary lesions. (a) Paired Ki‐67 index changes in the metastatic group. Each line connects primary and metastatic Ki‐67 values. Most patients showed increased proliferation. (b) Paired Ki‐67 index changes in the recurrent group. A more balanced distribution of increase, decrease, and stability was observed. (c) WHO grade shifts in the metastatic group. G1 → G2 and G2 → G3 transitions were the predominant patterns. (d) WHO grade shifts in the recurrent group. Only G1 → G2 progression occurred; no G2 → G3 changes were observed.

**Table S1:** Comparison of tumor grades between primary and metastatic/recurrent Lesions.
**Table S2:** Univariate analysis of factors associated with grade/Ki‐67 Index (≥ 10%) increase in metastatic lesions.
**Table S3:** Univariate analysis of factors associated with grade/Ki‐67 Index (≥ 10%) increase in recurrent lesions.

## Data Availability

The data that support the findings of this study are available on request from the corresponding author. The data are not publicly available due to privacy or ethical restrictions.
