# Supplementary figures and images for: Clinical Implications of Ki‐67 Index, Grade and Hormonal Changes in Pancreatic Neuroendocrine Tumors: Insights Into Tumor Heterogeneity Based on Primary and Secondary Lesions
Source: J Hepatobiliary Pancreat Sci. 2025 Nov 16;33(2):141–50. doi: 10.1002/jhbp.70028 (PMC12924110; doi:10.1002/jhbp.70028)

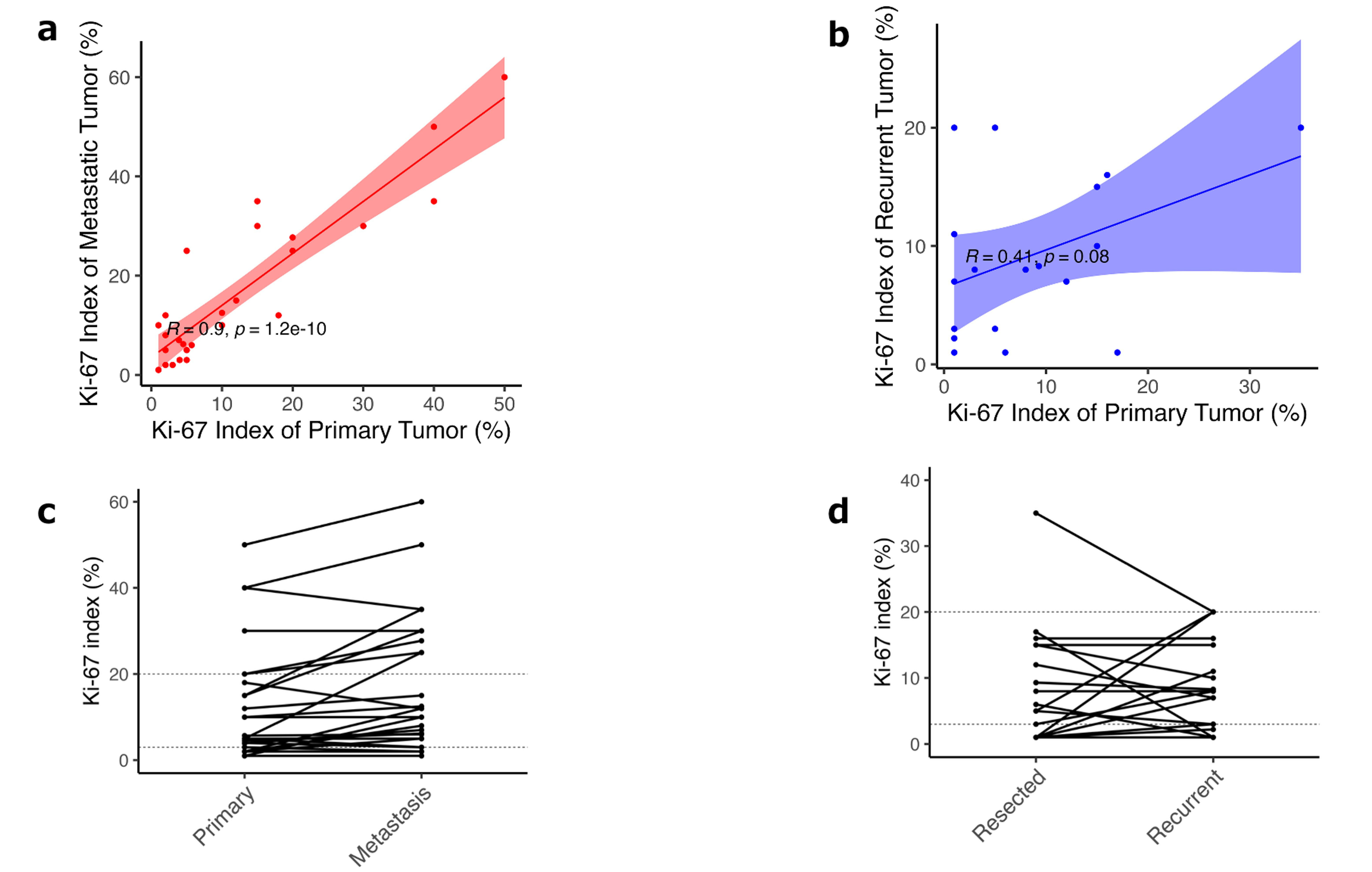

Supplement: Supplementary file 1 — Figure S1: Paired changes in Ki‐67 index and WHO grade between primary and secondary lesions. (a) Paired Ki‐67 index changes in the metastatic group. Each line connects primary and metastatic Ki‐67 values. Most patients showed increased proliferation. (b) Paired Ki‐67 index changes in the recurrent group. A more balanced distribution of increase, decrease, and stability was observed. (c) WHO grade shifts in the metastatic group. G1 → G2 and G2 → G3 transitions were the predominant patterns. (d) WHO grade shifts in the recurrent group. Only G1 → G2 progression occurred; no G2 → G3 changes were observed. [file JHBP-33-141-s001.jpg]
