# Supplementary material for: Clinical Implications of Ki‐67 Index, Grade and Hormonal Changes in Pancreatic Neuroendocrine Tumors: Insights Into Tumor Heterogeneity Based on Primary and Secondary Lesions
Source: J Hepatobiliary Pancreat Sci. 2025 Nov 16;33(2):141–50. doi: 10.1002/jhbp.70028 (PMC12924110; doi:10.1002/jhbp.70028)
Supplement: Supplementary file 2 — Table S1: Comparison of tumor grades between primary and metastatic/recurrent Lesions. Table S2: Univariate analysis of factors associated with grade/Ki‐67 Index (≥ 10%) increase in metastatic lesions. Table S3: Univariate analysis of factors associated with grade/Ki‐67 Index (≥ 10%) increase in recurrent lesions. [file JHBP-33-141-s002.docx]

| Supplementary Table 1. Comparison of tumor grades between primary and metastatic/ recurrent Lesions | | | | | |
| --- | --- | --- | --- | --- | --- |
|  | Secondary Grade | NET G1 | NET G2 | NET G3 | total |
| Primary Grade |  |  |  |  |  |
| Metastatic group |  |  |  |  |  |
| NET G1 |  | 2 | 4 | 0 | 6 |
| NET G2 |  | 1 | 11 | 5 | 17 |
| NET G3 |  | 0 | 0 | 4 | 4 |
| Recurrent Group |  |  |  |  |  |
| NET G1 |  | 2 | 4 | 0 | 6 |
| NET G2 |  | 2 | 10 | 0 | 12 |
| NET G3 |  | 0 | 1 | 0 | 1 |
| NET, neuroendocrine tumor. | | | | | |

| Supplementary Table 2. Univariate analysis of factors associated with grade/ Ki-67 Index (≥10%) increase in metastatic lesions | | | |
| --- | --- | --- | --- |
|  | Univariate | | |
| Characteristic | OR | 95% CI | p-value |
| Age at diagnosis | 1.04 | 0.98, 1.10 | 0.2 |
| SEX |  |  |  |
| Female | — | — |  |
| Men | 0.5 | 0.10, 2.36 | 0.4 |
| PS at diagnosis |  |  |  |
| PS0 | — | — |  |
| PS1 | 0.93 | 0.04, 11.5 | >0.9 |
| Primary tumor location |  |  |  |
| Pancreatic head | — | — |  |
| Other | 0.6 | 0.12, 3.00 | 0.5 |
| Functionality |  |  |  |
| Non-functioning | — | — |  |
| Functioning | 0.96 | 0.11, 7.00 | >0.9 |
| Primary tumor size | 0.98 | 0.94, 1.01 | 0.3 |
| Ki-67 index about primary lesion | 1.03 | 0.97, 1.11 | 0.3 |
| Grade about primary lesion |  |  |  |
| NET G1 | — | — |  |
| NET G2/G3 | 0.25 | 0.03, 1.60 | 0.2 |
| CI, confidence Interval; OR, odds ratio; PS, performance status; NET, neuroendocrine tumor. | | | |

| Supplementary Table 3. Univariate analysis of factors associated with grade/ Ki-67 Index (≥10%) increase in recurrent lesions | | | |
| --- | --- | --- | --- |
|  | Univariate | | |
| Characteristic | OR | 95% CI | p-value |
| Age at recurrence | 0.97 | 0.88, 1.06 | 0.6 |
| SEX |  |  |  |
| Female | — | — |  |
| Men | 1.2 | 0.13, 9.91 | 0.9 |
| PS at diagnosis |  |  |  |
| PS0 | — | — |  |
| PS1 | 0 |  | >0.9 |
| Primary tumor location |  |  |  |
| Pancreatic head | — | — |  |
| Other | 2 | 0.25, 19.2 | 0.5 |
| Functionality |  |  |  |
| Non-functioning | — | — |  |
| Functioning | 8.67 | 0.63, 229 | 0.12 |
| Primary tumor size | 0.91 | 0.78, 1.01 | 0.14 |
| Ki-67 index about resected lesion | 0.61 | 0.26, 0.90 | 0.093 |
| Grade about resected lesion |  |  |  |
| NET G1 | — | — |  |
| NET G2/G3 | 0.04 | 0.00, 0.41 | 0.019 |
| CI, confidence Interval; OR, odds ratio; PS, performance status; NET, neuroendocrine tumor. | | | |
